# College and Hospital Notes

**Published:** 1899-09

**Authors:** 


### COLLEGE AND HOSPITAL NOTES.

OFFICERS of the Pan-American Exposition Company have taken up the task of forming a medical bureau that will work while the exposition is being held. Nothing definite has yet been done and any plans that may have been formed are only tentative.

Omaha, with an attendance of nearly 3,000,000, had 3,095 sick cases within the exposition gates. The most of them were from exhaustion and minor causes, the number of serious cases being small.

Chicago, with an attendance more than ten times as great, had 11,602 cases of sickness on the grounds, but most of these, as at Omaha, were minor cases.

An examination shows that most of the cases at both places were those of exhaustion, people being overcome by heat, or old people simply tired out. The hospitals were not intended to accommodate patients for a great length of time, twenty-four hours being the longest period they were held.

It is proposed to build for the Pan-American Exposition a perfect hospital for temporary cases, all persons not discharged within twenty-four hours to be sent to some of the city hospitals. There will be a corps of nurses and doctors, just as in any other hospital, and all necessary appliances for such cases as will probably be treated. It is expected that there will be some accident cases, and an ambulance corps will be established.

Judging from Omaha and Chicago figures, there will be about 5,000 cases for the Pan-American hospital.—*Buffalo Evening News.*

